# Type 2 diabetes prevalence and its risk factors in HIV: A cross-sectional study

**DOI:** 10.1371/journal.pone.0194199

**Published:** 2018-03-12

**Authors:** Alastair D. Duncan, Louise M. Goff, Barry S. Peters

**Affiliations:** 1 HIV Medicine, Guy’s and St. Thomas’ Hospital NHS Trust, London, United Kingdom; 2 Diabetes and Nutritional Sciences, King’s College, London, United Kingdom; 3 Infection, Inflammation and Immunity, King’s College, London, United Kingdom; Istituto Di Ricerche Farmacologiche Mario Negri, ITALY

## Abstract

**Background:**

Type 2 diabetes (T2D) has a reported greater prevalence and poorer treatment outcomes in people living with HIV (PLWH) than comparable HIV-uninfected cohorts. We conducted a cross-sectional study to delineate the factors driving T2D in PLWH in an ethnically diverse cohort, and additionally observed how these have changed over time.

**Setting:**

We studied a diverse HIV cohort in London to determine the prevalence and risk factors for T2D, and compared them to a cohort studied 10 years previously.

**Methods:**

Patients were classified as normoglycaemic (fasting glucose <6.0 mmol/l) or dysglycaemic (≥6.0 mmol/l). The relative contribution to dysglycaemia of modifiable and fixed factors, including demographics, anthropometrics, comorbidities, immune status, and HIV therapy, were analysed using univariate and logistic regression analyses.

**Results:**

T2D prevalence was 15.1% in 2015 with a relative risk of 2.4 compared to the general population. The prevalence compared to 6.8% ten years earlier. The 2015 versus the 2005 cohort was significantly older (median age 49 (42–57) years versus 41 (IQR 35–47), p<0.001), had a higher BMI (27.4 (23.3–29.9) versus 24.9 (22.4–28.0) kg/m^2^ respectively, p = 0.019) and hypertensive (37.9% versus 19.6 respectively, p<0.001). The strongest predictors of dysglycaemia in the 2015 cohort were hepatic steatosis and hypertension, odds ratios (OR) and 95% confidence intervals (CI) 6.74 (3.48–13.03) and 2.92 (1.66–5.16) respectively, and also HIV-related factors of weight gain following antiretroviral initiation and longer known duration of HIV infection (OR 1.07 (1.04–1.11) and 1.06 (1.02–1.10) respectively).

**Conclusions:**

The alarmingly high prevalence of T2D in HIV requires improved screening, targeted to older patients and those with a longer duration of exposure to antiretrovirals. Effective diabetes prevention and management strategies are needed urgently to reduce this risk; such interventions should target both conventional risk factors, such as abdominal obesity, and HIV-specific risk factors such as weight gain following initiation of antiretrovirals.

## Introduction

Combination antiretroviral (ARV) therapy for people living with HIV (PLWH) has led to a marked reduction in HIV-associated morbidity and mortality since its introduction in the mid-1990s. Much of the disease burden in PLWH is now due to morbidities found in the general population. HIV itself, and some of the ARVs used to treat it, are associated with an increased risk and premature development of chronic comorbidities, including type 2 diabetes (T2D), which is reported to be up to four times more prevalent in PLWH than those without HIV [1,2].

Risk factors for T2D in the general population are well established [3] but in HIV there are additional specific risk factors, including duration of HIV infection, degree of immunosuppression, and exposure to those ARVs known to be associated with dysglycaemia [4,5]. Specific ARVs, with a variable contribution from HIV infection itself, are associated with an increased risk of metabolic diseases, including redistribution of fat and other manifestations of lipodystrophy, dyslipidaemia, and higher rates of comorbidities associated with ageing, including myocardial infarction and T2D [6]. Despite improvements in ARVs, the prevalence of conventional T2D risk factors appear to be increasing in HIV patients as the population ages [7]. It is important to understand the reasons for the increase in T2D in PLWH in order to effectively target early prevention strategies. In this cross-sectional study of an ethnically diverse cohort we aimed to undertake a detailed exploration of risk factors contributing to prediabetes and T2D, and to identify demographic and clinical differences over time.

## Methods

We recruited HIV patients from three London outpatient clinics serving an ethnically diverse HIV prevalent area. Data were collected at two time periods, 2005 and 2014/15. The 2005 cohort was studied as part of a substantive investigation of the metabolic profile of HIV [8]. The 2014–2015 cohort participants were recruited to enable an in-depth examination of factors associated with risk of type 2 diabetes, and secondarily to facilitate a comparison over time. Research Ethics Committee approval was granted (UK Health Research Authority reference 13 LO 1543) and participants gave written informed consent.

For the 2005 cohort the approach to random selection of clinic attendees and data collection has been described previously [9]. In summary, every third patient attending outpatients was invited to participate, with recruitment from specialist metabolic clinics excluded. Patients attending these specialist clinics could be recruited from general attendance. For the present analyses, the subset of participants for whom fasting blood glucose data were available were included (n = 337), 33.0% of the total cohort. Sensitivity analyses were conducted to compare the subset of participants with the full cohort; no significant differences were found in demographic, medical or HIV characteristics of the cohorts (S1 Table).

The 2014/15 cohort was recruited using structured random sampling. A 48-cell grid stratified by ten-year age bands, gender and ethnicity (White, Black African, Black Caribbean, Other) was constructed to exactly mirror the demographic spread for those parameters of all HIV patients attending the clinics. We aimed to ensure the diversity of the cohort was represented as women and minority ethnic groups are routinely underrepresented in clinical research [10]. Recruitment was halted to each cell on achievement of individual targets. Participants were recruited through a combination of self-referral in response to within-clinic advertising, sampling of every third clinic attendee, and by healthcare professional referral. All patients were eligible to participate except those unable to provide informed consent or communicate adequately during study procedures, or those serving custodial sentences. Sample size was calculated using assumptions from the 2005 data indicating a prevalence of dysglycaemia of 25% with a mean BMI of 25.8 kg/m^2^. With absolute and type 1 errors both set at 5% the sample size was calculated to be 339.

In both cohorts each participant attended a research data collection visit and information was collected from medical record review. At the assessment visit data collected prospectively included: body mass index (BMI) measured using calibrated electronic weighing scales and stadiometer, and categorised as underweight (<18.5 kg/m^2^), normal (18.5–24.9 kg/m^2^), overweight (25.0–29.9 kg/m^2^) and obese (≥30.0 kg/m^2^) [11]; waist measured using a non-stretch tape and categorised as normal or centrally obese using International Diabetes Federation (IDF) criteria [11]; hypertension determined as the mean of three blood pressure measurements greater than 140/80 mm Hg (measured by electronic sphygmomanometer) or current antihypertensive use [12]; smoking status categorised as current, never, or ex-smoker; serum vitamin D concentration (defined as most recent within 3 years of data collection); 10-year cardiovascular (CVD) risk calculated using the Framingham tool [13]; and dyslipidaemia (defined as total cholesterol, LDL cholesterol or triglycerides above 5.2 mmol/l, 3.0 mmol/l and 2.2 mmol/l respectively, or HDL cholesterol below 1.2 mmol/l, measured from fasting phlebotomy at the visit. All participants attended after an overnight 10–12 hour fast for assessment of fasting plasma glucose, and were stratified according to glycaemic status by either a recorded diagnosis of T2D, or a confirmed fasting glucose: normal (≤5.9 mmol/l), impaired fasting glucose (6.0–6.9 mmol/l), and T2D (≥7.0 mmol/l). Additionally at the research visit the following variables were recorded from the medical notes and verified by the participant: age; gender; ethnicity; history of CVD; duration of HIV infection; current and historic ARV type and duration of treatment; current HIV suppression; lipodystrophy defined as current or historic by participant recall and/or physician diagnosis from the medical notes; and current hepatitis co-infection (successful clearance of Hepatitis C categorised as negative). All blood samples were analysed in the same laboratory for consistency. Data were anonymised.

In 2015 the data collection was extended to include a broader range of risk factors including variables assessed from the medical notes and verified by the participant at the research visit: intake of fruits and vegetables (five daily portions is the national guideline intake [14]); weekly hours of physical activity (exertion greater than usual walking pace); history of CVD and stroke recorded separately; family history of diabetes; current chronic kidney disease; and historic weight change in the 12 months following initiation of ARVs. Additional variables were assessed from the medical notes including: current hepatic steatosis assessed using standard clinical definitions by biopsy, MRI scan, or assessed by Fibroscan with an attenuation greater than 250 [15]; and CD4 nadir.

The following ARVs were categorised as most associated with the development of insulin resistance, according to a review of published literature: zidovudine, didanosine, stavudine, zalcitabine, indinavir, and high-dose ritonavir [1,4,16,17]. Metabolic syndrome was defined using IDF criteria [11]. Known duration of HIV infection and of ARV therapy were measured from the dates of the first known positive HIV antibody test and first use of any ARV, respectively.

### Data analysis

Data were analysed using IBM SPSS, version 22. Continuous variables were checked for normality. All variables were categorised as fixed other than these categorised as modifiable: BMI, waist, hypertension, dyslipidaemia, dietary intake, physical activity, and hepatic steatosis. Characteristics of the 2005 and 2015 cohorts were compared, and differences between the two were calculated and assessed for statistical significance using Chi-squared and ANOVA tests. For the 2015 cohort, the relative risk of developing diabetes compared to the general population was estimated using the QDiabetes score [18]. Patients with prediabetes (impaired fasting glucose) and T2D were combined into a single group, ‘dysglycaemia’, and differences between normal and dysglycaemia were analysed using Chi-squared and ANOVA tests. Significantly co-linear variables were excluded from further analysis. Variables significantly associated with dysglycaemia were used to construct multiple logistic regression models with backwards stepwise removal. Modifiable and fixed factors were modelled separately, and finally all factors were modelled together to estimate relative odds ratio (OR) contributions to predict dysglycaemia.

## Results

### Cohort characteristics

The 2005 cohort consisted of 337 participants (77% male) and the 2015 cohort consisted of 338 participants (74% male) (S1 Fig). The clinical characteristics of the cohorts are shown (Table 1). Both cohorts were ethnically diverse, with 61 countries of birth documented in 2015 reflecting the diversity of the wider London population. In 2015 58% of participants were overweight or obese. Compared to 2005, the 2015 cohort was older, heavier, more hypertensive, had been HIV positive for longer, and were more likely to have been treated with ARVs, but had lower rates of smoking and lipodystrophy. Participants in this study experienced no adverse events. There was no missing data other than a lack of vitamin D measurement for 138 participants.

**Table 1:** Clinical characteristics of the 2005 and 2015 cohorts.

		2005 (n = 337)	2015 (n = 338)	*p*
GENDER	Male	77.2%	74.0%	0.335^a^
AGE (Years)	Median (IQR)	41 (*35–47)*	49 *(42–57)*	<0.001^b^
ETHNICITY	White	54.6%	49.7%	0.525^a^
	Black African	28.2%	31.7%	
	Black Caribbean	5.9%	7.7%	
	Other	11.3%	10.9%	
TYPE 2 DIABETES		6.8%	15.1%	0.003^a^
BMI (kg/m^2^)	Median (IQR)	24.9 *(22*.*4–28*.*0)*	27.4 *(23*.*3–29*.*9)*	0.019^b^
WAIST (IDF DEFINITION)	Obese	47.7%	62.4%	0.024^a^
(cm)	Median (IQR)	91 *(83–98)*	95 *(86–104)*	0.055^b^
HYPERTENSION	%	19.6%	37.9%	<0.001^a^
LIPIDS (mmol/l)	Total Cholesterol (SD)	4.8 *±1*.*0*	5.0 *±1*.*1*	0.242^b^
	HDL: TG Ratio (SD)	1.59 *±1*.*74*	1.39 *±1*.*19*	0.180^b^
METABOLIC SYNDROME (IDF DEFINITION)	%	22.5%	31.8%	<0.001^b^
SMOKING	Current	35.6%	21.0%	0.019^a^
CARDIOVASCULAR DISEASE	(includes stroke)	2.7%	5.6%	0.055^a^
STATIN USE	Current	16.0%	27.2%	<0.001^a^
% 10-YEAR CVD RISK (Framingham)	Mean (SD)	4.4 *±5*.*4*	12.1 *±12*.*3*	<0.001^b^
HIV Duration (Years)	Mean (SD)	6.3 *±0*.*9*	11.6 *±0*.*9*	<0.001^b^
ANTIRETROVIRALS (ARVs)	Naïve	19.6%	8.0%	<0.001^a^
ARVs ASSOCIATED WITH T2D	Current or historic	23.4%	44.1%	<0.001^a^
LIPODYSTROPHY	Current or historic	27.3%	21.6%	0.007^a^
HEPATITIS B	Current	4.5%	9.2%	0.021^a^
HEPATITIS C	Current	3.6%	4.7%	0.433^a^

^a^ Difference between 2005 and 2015 by Chi-squared or Fishers-exact tests;

^b^ Difference between 2005 and 2015 by ANOVA test.

ARVs: Antiretrovirals; BMI: Body Mass Index; CVD: Cardiovascular Disease; HDL: High Density Lipoprotein Cholesterol; IDF: International Diabetes Federation; IQR: Interquartile Range; SD: Standard Deviation; TG: Triglycerides.

### Dysglycaemia

The prevalence of dysglycaemia was 24.9% in 2005 compared to 32.3% in 2015. The prevalence of impaired fasting glucose excluding T2D did not differ between the cohorts (2005: 18.1 *vs* 2015: 17.2%, *p* = 0.763). However, the prevalence of T2D was significantly higher in 2015 compared to 2005 (2005: 6.8 vs 2015: 15.1%, *p* = 0.003). In the 2015 cohort, using the QDiabetes Score the relative risk of T2D was estimated at 2.4. Factors associated with dysglycaemia are shown in Table 2. Those significantly associated with dysglycaemia at both time points included age and waist circumference (2005 and 2015, *p*<0.001), hypertension (2005 *p* = 0.001; 2015 *p*<0.001), duration of HIV infection (2005 *p* = 0.046; 2015 *p*<0.001) and the use of ARVs (2005 *p* = 0.018; 2015 *p* = 0.009). Exposure to ARVs associated with diabetes and BMI were both significantly correlated in 2015 only (2005 *p* = 0.878; 2015 *p* = 0.002; *p* = 0.423 and *p* = 0.001 respectively). Factors measured only in 2015 significantly associated with dysglycaemia were hepatic steatosis, low levels of physical activity and weight gain following initiation of ARVs (*p*<0.001 for all).

**Table 2:** Risk factors associated with dysglycaemia in the 2005 and 2015 cohorts.

		2005		2015	
		*r*	*p*	*r*	*p*
GENDER		0.02	0.721^a^	0.03	0.311^a^
AGE	Median Years	0.19	<0.001^b^	0.60	<0.001^b^
ETHNICITY	White	0.07	0.325^a^	0.08	0.531^a^
	Black African	0.06	0.506^a^	0.08	0.358^a^
	Black Caribbean	0.10	0.108^a^	0.06	0.253^a^
BMI^d^	Median (kg/m2)	0.04	0.423^b^	0.17	0.001^b^
WAIST^d^	Median	0.24	<0.001^b^	0.27	<0.001^b^
	IDF Definition	0.30	<0.001^a^	0.26	<0.001^a^
HYPERTENSION	Current	0.18	0.001^a^	0.39	<0.001^a^
LIPIDS	Total Cholesterol	0.09	0.118^b^	0.05	0.438^b^
	HDL	0.05	0.335^b^	0.13	0.016^b^
	LDL^c^			0.10	0.072^b^
	Triglycerides^d^	0.11	0.038^b^	0.30	<0.001^b^
	HDL:TG Ratio	0.08	0.130^b^	0.27	<0.001^b^
METABOLIC SYNDROME	Current	0.58	<0.001^a^	0.63	<0.001^a^
SMOKING	Current	0.01	0.716^a^	0.12	0.905^a^
CVD (excluding stroke)	Current or historic	0.10	0.170^a^	0.11	0.050^a^
STATIN USE	Current	0.14	0.043^a^	0.40	<0.001^a^
STROKE	Current or historic			0.10	0.109^a^
% 10-YEAR CVD RISK	Framingham, mean	0.07	0.210^b^	0.39	<0.001^b^
HIV Duration	Mean Years	0.11	0.046^b^	0.18	<0.001^b^
ARV	Treated	0.13	0.018^a^	0.17	0.009^a^
ARVs ASSOCIATED WITH DYSGLYCAEMIA		0.01	0.878^a^	0.16	0.002^a^
PERCENTAGE WEIGHT GAIN IN YEAR FOLLOWING INITITATION OF ARVs^c^				0.34	<0.001^b^
LIPODYSTROPHY	Current or historic	0.20	0.061^a^	0.15	0.007^a^
HEPATITIS B	Current	0.01	0.873^a^	0.04	0.421^a^
HEPATITIS C	Current	0.09	0.170^a^	0.01	0.930^a^
CD4 NADIR^c^	Mean			0.01	0.082^b^
VITAMIN D^c^	Mean			0.11	0.134^b^
HEPATIC STEATOSIS^c^	Current			0.45	<0.001^a^
CHRONIC KIDNEY DISEASE^c^	Current			0.10	0.073^a^
CORTICOSTEROID THERAPY^c^	Current or historic			0.14	0.014^a^
1st or 2nd DEGREE RELATIVE WITH T2D^c^				0.10	0.076^a^
PHYSICAL ACTIVITY^c^	Hours per week			0.20	<0.001^b^
FRUITS & VEGETABLES^c^	Portions per day			0.06	0.260^b^

Participants grouped as normal or dysglycaemia (impaired fasting glucose and type 2 diabetes)

*r* = correlation coefficient; *p* = significance.

^*a*^ Significance by Pearson Chi-squared test with Phi correlation;

^b^ Significance by Pearson Correlation;

^c^ Data not collected in 2005;

^d^ Non-normal distribution log-transformed

We conducted logistic regression analysis, modelling factors associated with dysglycaemia in our 2015 cohort. Our first model examined the predictive nature of modifiable risk factors, the second looked at fixed factors and the final model examined all factors. All three models showed statistical significance (all *p*<0.001) (Table 3). Modifiable factors (hepatic steatosis, hypertension, HDL: triglyceride ratio and physical activity) predicted dysglycaemia more strongly than fixed and HIV-related factors (age, duration of HIV infection and weight gain following initiation of ARVs), with hepatic steatosis (OR 7.28, *p*<0.001) and hypertension (OR 2.58, *p* = 0.003) the strongest predictors.

**Table 3:** Logistic regression analysis of factors predictive of dysglycaemia in the 2015 cohort.

	Adjusted Odds Ratio (95% CI)					
	*Model 1*: *Modifiable Factors* *(n = 336)*	*Factor* *p-value*	*Model 2*: *Fixed Factors* *(n = 294)*	*Factor* *p-value*	*Model 3*: *All Factors* *(n = 293)*	*Factor* *p-value*
**Physical Activity** ^a^ **(hours)**	0.91 (0.82–1.00)	0.042				
**Hypertension** ^b^	2.92 (1.66–5.16)	<0.001			2.58 (1.37–4.88)	0.003
**Hepatic Steatosis** ^b^	6.74 (3.48–13.03)	<0.001			7.28 (3.46–15.34)	<0.001
**HDL: Triglyceride Ratio** ^a^ **(mmol/l)**	1.56 (1.22–1.99)	<0.001			1.73 (1.32–2.27)	<0.001
**% Weight Gain after ARVs** ^a^			1.07 (1.04–1.11)	<0.001	1.06 (1.02–1.10)	0.003
**Age** ^a^ **(years)**			1.06 (1.03–1.09)	<0.001	1.07 (1.03–1.10)	<0.001
**Duration of HIV Infection** ^a^ **(years)**			1.06 (1.02–1.10)	0.003		

Collinearity was observed between the variables waist and BMI (Pearson correlation 0.851) and between HIV Duration and CD4 Nadir (Pearson correlation 0.365), and the latter of each pair was excluded from regression modelling.

Continuous variables:

^a^; binary variables:

^b^Model Chi-square values: Model 1, 107.21 (*p*<0.001); model 2, 53.14 (*p*<0.001); model 3, 121.69 (*p*<0.001).

In Models 2 and 3 reduced participant numbers are due to exclusion of those who were not treated with ARVs or who had not yet received 12 month’s ARV treatment in order to calculate percentage weight gain in that time.

## Discussion

In this ethnically diverse cohort of HIV patients we have shown that there is an alarmingly high prevalence of dysglycaemia: approximately 1 in 3 patients have prediabetes or T2D. We have determined the role of a range of both HIV-specific and conventional risk factors in predicting the prevalence of T2D, and demonstrated the importance of modifiable risk factors. Compared to 2005 the 2015 cohort were older, had longer duration of HIV status and greater exposure to ART therapy, all significant determinants of dysglycaemia. However, our analysis also recognised the importance of a number of other determinants such as central adiposity which were more prevalent in the 2015 cohort. Given the burden of T2D in PLWH there is an urgent need to mitigate these modifiable risk factors through intervention in terms of both prevention and treatment.

The key risk factors for T2D in the general population, body mass and waist circumference, disproportionately affect people from minority ethnic groups [19], and these risk factors are pronounced in this ethnically diverse cohort. Historically HIV was a disease associated with wasting and premature death and therefore these conventional risk factors were of little relevance. However, PLWH are now living longer and we have demonstrated that the rates of overweight and abdominal obesity are increasing and are now comparable with the general population [20]. Our regression models help explain the rise in T2D prevalence in our cohort, with changes in the incidence of conventional risk factors combined with HIV-specific factors contributing to T2D risk. The duration of HIV infection, ARV treatment and particularly the use of metabolically toxic ARVs, weight gain following initiation of ARVs, and the presence of lipodystrophy are all significantly associated with an increased risk of dysglycaemia. These were more likely to affect the 2015 cohort than in 2005, apart from lipodystrophy for which we observed a lower prevalence. Modifiable factors contributed significantly more to risk of dysglycaemia in our cohort than fixed factors, including age. In our cohort, hepatic steatosis and hypertension were the strongest predictors of dysglycaemia. HIV-specific factors also contributed significantly, particularly weight gain following initiation of ARVs, mirroring other recent observations [21]. It is not known if lifestyle treatment of hepatic steatosis directly reduces risk of development of diabetes, however pharmacological treatment of hypertension has been associated with a reduced risk for diabetes in the general population. Given our findings suggesting a link between both hepatic steatosis and hypertension with diabetes risk, we believe that further research is required in people living with HIV.

The prevalence of T2D in the 2005 cohort was 6.8% whilst in our 2015 cohort it was 15.1%, driven in part by increasing age and BMI. Few studies elsewhere have examined change in prevalence of T2D in PLWH, however a change in prevalence of T2D was observed in an ethnically diverse HIV cohort in France [22]. A rise in incidence was associated with age and obesity, and historic exposure to ARVs linked with metabolic toxicities, as observed in the study presented here. Conversely, a study of a Danish population showed no increased risk for T2D in HIV, however this study was confined to White participants, the cohort was relatively young, and had lower levels of obesity [17]. Excess mortality when BMI is above normal has been well-demonstrated in the general population [23], and is of concern in PLWH: in one US cohort, multimorbidity associated with obesity affected 65% of HIV patients [24]. The prevalence of T2D in our 2015 cohort exceeded the upper end of a range in PLWH that has been described as 2.6–14% [1]. This may in part reflect collection of our data as recently as 2015, and might be a portent of a more general trend, especially given the ageing nature of the cohort. Our high prevalence might also reflect socioeconomic challenges faced by a high proportion of our largely inner city cohort. It is unlikely that the difference in sampling strategies between the cohorts contributed to the high prevalence in 2015 as although Black Caribbean and Black African participants were fairly represented in 2015, the difference to 2005 was not statistically significant.

It is well established that dietary change, increased physical activity and weight loss all reduce diabetes risk in the general population [25]. This approach may be especially relevant to PLWH given the low levels of physical activity and fruit and vegetable consumption and high prevalence of overweight and obesity that we observed [20]. Dietetic intervention in PLWH has been shown to prevent weight gain and subsequent development of hyperlipidaemia after initiation of ARVs [26]. The association between dysglycaemia and weight gain following initiation of ARVs that we observed is another rationale for recommending dietetic intervention at this stage.

The strengths and limitations of our research warrant consideration. A principle strength for investigation of factors associated with diabetes risk in the 2015 cohort is the lack of missing data other than Vitamin D measurements. Our participants were recruited from the same clinical cohort at two time points, and this allowed us to compare factors associated with diabetes risk. Clinical definitions and diagnostic criteria were applied consistently in both 2005 and 2015. At both time points glycaemic status was characterised by glucose measured in a truly fasting state, and did not rely on HbA1c, which can be underestimated in people treated with ARVs [27].

A limitation is that our sampling methods differed between the two cohorts, although both included partial randomisation and excluded recruitment from specialist metabolic HIV clinics. It is possible that the 2015 sample does not fairly represent the total clinical cohort considering factors other than age, gender and ethnicity, for example socioeconomic status or educational attainment. Fewer predictor variables were collected in 2005 compared to 2015. Data collection from 2005 was not designed to allow subgroup analysis of factors contributing to diabetes risk, and so changes over time cannot be assessed. Regarding potential non-ARV pharmacological contribution to dysglycaemia, data collection was limited to exposure to corticosteroids and metformin. Furthermore, medical records predating 1996 were rarely available, and we relied on participant recollection which may not have been wholly accurate. Smoking status, physical activity levels and consumption of fruits and vegetables were self-reported and open to misreporting. Hepatic steatosis rates derived from the medical notes may have been overestimated in those with dysglycaemia due to selective screening in those perceived to be at risk of hepatic fibrosis. A limitation is the lack of an HIV negative control group for comparison.

In conclusion, the alarmingly high prevalence of T2D in our cohort of PLWH has implications for the tens of millions of PLWH worldwide. This is compounded by an increase in associated comorbidities in PLWH, including cardiovascular disease [6]. Additionally there are distinct challenges associated with the management of T2D in HIV. Some ARVs can impair glycosylated haemoglobin (HbA1c) assays resulting in underestimation [27,28] and some ARVs significantly interact with hypoglycaemic agents [29]. Overall it is known that HIV patients with T2D have a poorer response to diabetes treatments compared to matched HIV negative individuals [30]. We suggest a response is urgently needed. Enhanced screening for diabetes risk in PLWH should be considered and should account for the broad range of risk factors that affect PLWH, both conventional and HIV-specific. Interventions that tackle these risk factors are needed. Selection of ARVs with less effect on insulin resistance in those with dysglycaemia or significant risk factors, and proactive lifestyle management of the increased risk of diabetes that occurs due to weight gain following initiation of HAART, should be considered. Furthermore, there is an urgent need to understand whether diet and lifestyle interventions that are effective at tackling diabetes risk factors in the general population, for example those that reduce blood pressure and hepatic steatosis, are effective in PLWH. Diabetes risk should not be considered in isolation in PLWH, and disease specific screening and management of other comorbidities in PLWH with diabetes should be undertaken [1,31].

## Supporting information

S1 Table Sensitivity analysis for the 2005 sample (n = 337) compared with the total cohort (n = 1021). (PDF)

S1 Fig Consort diagram for recruitment of participants in 2015. (PDF)
