# Supplementary material for: Type 2 diabetes prevalence and its risk factors in HIV: A cross-sectional study
Source: PLoS One. 2018 Mar 12;13(3):e0194199. doi: 10.1371/journal.pone.0194199 (PMC5847234; doi:10.1371/journal.pone.0194199)
Supplement: S1 Table — (PDF) [file pone.0194199.s001.pdf]

S1 Table. Sensitivity Analysis for 2005 Sample (n=337) Compared With Total Cohort (n=1021).

		†2005 SAMPLE n=337	††TOTAL COHORT n=1021	*p*
TOTAL	n	337	1021	
GENDER	Male	260 (77.2%)	764 (74.8%)	0.265[a]
AGE	Mean years	41.7	40.4	0.134[b]
ETHNICITY	White	184 (54.6%)	512 (50.1%)	0.407[a]
BMI	Mean kg/m^2	25.8	25.5	0.835[b]
WAIST	Mean cm	91.3	90.1	0.132[b]
HYPERTENSION	n	66 (19.6%)	192 (18.8%)	0.891[a]
LIPIDS (mean)	Total Cholesterol	4.8	4.7	0.794[b]
mmol/l	Triglycerides	1.8	1.7	0.237[b]
CURRENT SMOKER	n	120 (35.6%)	378 (37.0%)	0.326[a]
CVD	n	9 (2.7%)	32 (3.1%)	0.563[a]
STATIN USE	n	54 (16.0%)	129 (12.6%)	0.268[a]
HIV DURATION	Mean years	6.3	6.5	0.249[b]
ARV NAIVE	n	66 (19.6%)	223 (21.8%)	0.426[a]
LIPODYSTROPHY	n	92 (27.3%)	216 (21.2%)	0.336[a]
HEPATITIS B	n	15 (4.5%)	50 (4.9%)	0.981[a]
HEPATITIS C	n	12 (3.6%)	34 (3.3%)	0.831[a]

Notes:

† Participants where glycaemia was measured

††Total sample including where glycaemia was not measured

[a] Significance of difference by Chi-squared

[b] Significance of difference by ANOVA
