# Supplementary figures and images for: Type 2 diabetes prevalence and its risk factors in HIV: A cross-sectional study
Source: PLoS One. 2018 Mar 12;13(3):e0194199. doi: 10.1371/journal.pone.0194199 (PMC5847234; doi:10.1371/journal.pone.0194199)

**S1 Fig. Consort Diagram for Recruitment of Participants in 2015.**

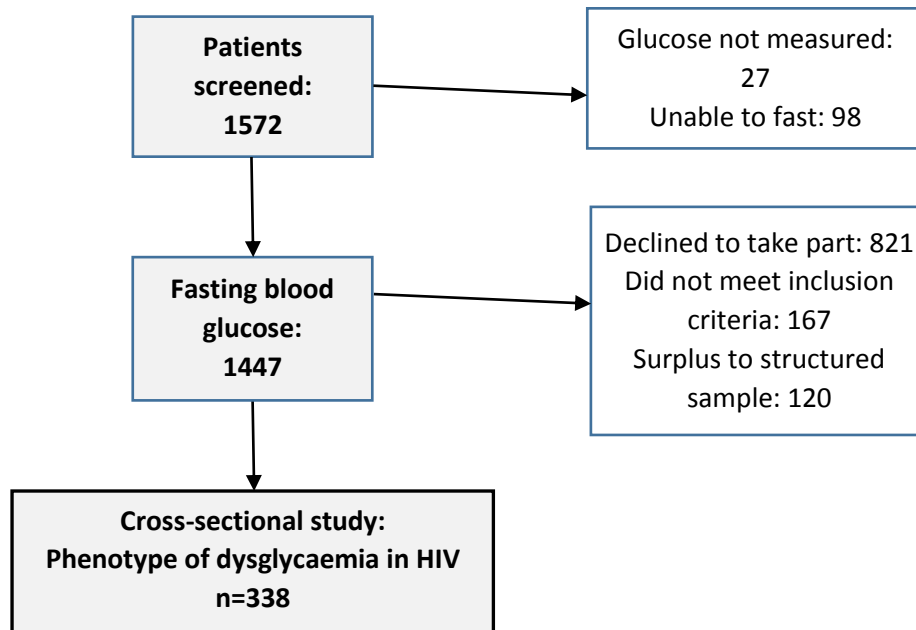

Supplement: S1 Fig — (PDF) [file pone.0194199.s002.pdf]
